# RGC-32 Regulates Generation of Reactive Astrocytes in Experimental Autoimmune Encephalomyelitis

**DOI:** 10.3389/fimmu.2020.608294

**Published:** 2021-01-25

**Authors:** Alexandru Tatomir, Austin Beltrand, Vinh Nguyen, Dallas Boodhoo, Armugam Mekala, Cornelia Cudrici, Tudor C. Badea, Dafin F. Muresanu, Violeta Rus, Horea Rus

**Affiliations:** ^1^ Department of Neurology, University of Maryland, School of Medicine, Baltimore, MD, United States; ^2^ Department of Neurosciences, “Iuliu Hatieganu” University of Medicine and Pharmacy, Cluj-Napoca, Romania; ^3^ Department of Medicine, Division of Rheumatology and Clinical Immunology, University of Maryland, School of Medicine, Baltimore, MD, United States; ^4^ Translational Vascular Medicine Branch, National Heart, Lung and Blood Institute, National Institutes of Health, Bethesda, MD, United States; ^5^ Retinal Circuit Development and Genetics Unit, Neurobiology Neurodegeneration & Repair Laboratory (N-NRL), National Eye Institute, Bethesda, MD, United States; ^6^ Research Service, Veterans Administration Maryland Health Care System, Baltimore, MD, United States

**Keywords:** Response Gene to Complement-32, astrocyte, radial glia, experimental autoimmune encephalomyelitis, GFAP, vimentin, FABP7

## Abstract

Astrocytes are increasingly recognized as critical contributors to multiple sclerosis pathogenesis. We have previously shown that lack of Response Gene to Complement 32 (RGC-32) alters astrocyte morphology in the spinal cord at the peak of experimental autoimmune encephalomyelitis (EAE), suggesting a role for RGC-32 in astrocyte differentiation. In this study, we analyzed the expression and distribution of astrocytes and astrocyte progenitors by immunohistochemistry in spinal cords of wild-type (WT) and RGC-32-knockout (KO) mice with EAE and of normal adult mice. Our analysis showed that during acute EAE, WT astrocytes had a reactive morphology and increased GFAP expression, whereas RGC-32 KO astrocytes had a morphology similar to that of radial glia and an increased expression of progenitor markers such as vimentin and fatty acid binding protein 7 (FABP7). In control mice, GFAP expression and astrocyte density were also significantly higher in the WT group, whereas the number of vimentin and FABP7-positive radial glia was significantly higher in the RGC-32 KO group. *In vitro* studies on cultured neonatal astrocytes from WT and RGC-32 KO mice showed that RGC-32 regulates a complex array of molecular networks pertaining to signal transduction, growth factor expression and secretion, and extracellular matrix (ECM) remodeling. Among the most differentially expressed factors were insulin-like growth factor 1 (IGF1), insulin-like growth factor binding proteins (IGFBPs), and connective tissue growth factor (CTGF); their expression was downregulated in RGC-32-depleted astrocytes. The nuclear translocation of STAT3, a transcription factor critical for astrogliogenesis and driving glial scar formation, was also impaired after RGC-32 silencing. Taken together, these data suggest that RGC-32 is an important regulator of astrocyte differentiation during EAE and that in the absence of RGC-32, astrocytes are unable to fully mature and become reactive astrocytes.

## Introduction

Response Gene to Complement (RGC)-32 was first identified in rat oligodendrocytes as a novel gene induced by complement activation (1). Subsequent studies have shown that RGC-32 is an important cell cycle regulator, driving cell cycle progression in a number of cell types, such as smooth muscle cells and endothelial cells, by promoting the activity of CDC2/cyclin B complexes and Akt kinase (2, 3). RGC-32 has also been shown to be involved in cell differentiation, tumorigenesis, and wound healing (4, 5). Furthermore, RGC-32 has been found to be crucial for the TGF-β-induced epithelial-to-mesenchymal transition in renal fibrosis and cancer metastasis (6–8).

Astrocytes are the most abundant glial cells in the central nervous system (CNS). They play an important role in maintaining CNS homeostasis and neuronal functions, contributing to synaptogenesis and neuronal plasticity, neurotransmitter clearance from the synaptic space, water and ionic balance, formation of the blood–brain barrier (BBB) through their end feet processes, and regulation of cerebral blood flow (9–11). Astrocytes were initially viewed as passive bystanders in CNS diseases, but their ability to undergo complex morphological and molecular changes in response to CNS damage has been increasingly recognized (12). This complex set of changes is collectively termed reactive astrogliosis (13, 14). Reactive astrocytes are now widely regarded as critical players in the pathophysiology of CNS diseases. Together with neurons and other glial cells, reactive astrocytes sustain neuroinflammation and dictate the outcome, either toward recovery and resolution of the injury or toward permanent damage and disease progression (15). This relatively delicate balance depends mostly on the heterogeneous nature of the reactive astrocytes and is time-, region-, and context-dependent.

The dual role of reactive astrocytes in the pathogenesis of multiple sclerosis (MS), a chronic, inflammatory demyelinating disease of the CNS, has gained considerable attention *via* studies in MS patients and in its animal model, experimental autoimmune encephalomyelitis (EAE) (15, 16). Elegant studies have demonstrated that reactive astrocytes can sustain disease initiation and progression through multiple mechanisms, including secretion of inflammatory cytokines and chemokines that recruit inflammatory cells to the CNS, production of reactive oxygen and nitrogen species, disruption of the BBB, facilitation of demyelination, and inhibition of remyelination (15–19). Recently, a highly neurotoxic phenotype of reactive astrocytes termed A1 was described and shown to be present in MS lesions (20). On the other hand, reactive astrocytes also have beneficial effects and can help to stop disease progression and promote recovery. These beneficial effects in limiting inflammation rely on the cells’ ability to form the glial scar, which corrals inflammatory infiltrates and prevents their spread to adjacent tissue (21, 22). Reactive astrocytes can also polarize immune cells toward a more regulatory and anti-inflammatory phenotype and can promote remyelination by stimulating migration, proliferation, and differentiation of oligodendrocyte precursor cells (16). Despite these protective effects, it is thought that the overall effect of reactive astrogliosis may be detrimental, given the numerous studies that have shown a positive correlation between disease severity and the degree of astrocytic activation. Reactive astrocytes are now seen as a major contributor to MS progression (15).

We have previously demonstrated that mice with EAE that lack RGC-32 develop a less severe disease course when compared with their WT counterparts (23). RGC-32 KO mice show a significant reduction in the amount of inflammatory infiltrate, with fewer IL-17-positive as well as granulocyte-macrophage colony-stimulating factor (GM-CSF)-positive cells, which play a crucial role in the induction and evolution of EAE (23). *In vitro* studies have shown that RGC-32 is critical for the differentiation of naïve T cells toward the Th17 phenotype (23, 24). Moreover, we have found that the expression of collagens type I, IV, and V and fibronectin, together with that of the reactive astrocyte markers nestin and alpha-smooth muscle actin, is decreased in cultured astrocytes after siRNA-mediated RGC-32 silencing and in astrocytes purified from RGC-32-KO mice, suggesting that RGC-32 facilitates the TGF-β-induced expression of these components (25). These effects are mediated at the molecular level by RGC-32’s interaction with SMAD3 and the nuclear translocation of the two interacting molecules, which plays a central role in the intracellular signaling pathway downstream of the TGF-β receptor (25). Interestingly, we have also observed that there are differences in the morphology of spinal cord astrocytes between WT and RGC-32 KO mice with EAE (25), but whether RGC-32 is involved in regulating astrocyte differentiation *in vivo* during EAE is not yet known. Here, we show that in the absence of RGC-32, spinal cord astrocytes have a lower density and display an immature phenotype associated with increased expression of the radial glial markers vimentin and fatty acid binding protein 7 (FABP7) and, in contrast to WT astrocytes, they are unable to undergo reactive changes at the peak of EAE disease. We also show that mechanistically, RGC-32 regulates a complex array of molecular networks related to signal transduction, growth factor expression and secretion, and extracellular matrix (ECM) remodeling. Among these factors, we found the most differentially expressed to be insulin-like growth factor 1 (IGF1), insulin-like growth factor binding proteins (IGFBPs), and connective tissue growth factor (CTGF), with the expression of these proteins being downregulated in RGC-32-depleted astrocytes. RGC-32 apparently also regulates the nuclear translocation of STAT3, a transcription factor critical for the gliogenic switch early in astrogliogenesis and for glial scar formation, since RGC-32 silencing in cultured astrocytes impairs the nuclear translocation of STAT3. These results suggest that RGC-32 is an important mediator of astrocyte differentiation and that RGC-32 KO astrocytes are unable to undergo reactive changes during EAE because of their immature phenotype, consistent with the protective effects seen in RGC-32 KO mice.

## Materials and Methods

### Mice

All mice were on a C57BL/6 background, used at 6–12 weeks of age, and housed under specific pathogen-free conditions. The generation of RGC-32 KO mice has been described previously (26). WT C57BL/6 littermates were used as controls. All procedures were approved by the University of Maryland School of Medicine Office of Animal Welfare Assurance.

### Induction and Evaluation of EAE

RGC-32 KO and WT female mice (8–10 weeks old) were injected subcutaneously in two locations in the dorsal flank with an emulsion containing 200 μg of MOG_35–55_ (Anaspec, Fremont, CA) and complete Freund’s adjuvant (CFA) (Difco, Detroit, MI) as previously described (23). Pertussis toxin (400 ng; List Biological Laboratories, Campbell, CA) was administered intraperitoneally on days 0 and 2. Mice were monitored daily, and the disease was scored on a scale of 0–5 as follows: 1—limp tail; 2—hindlimb paresis; 3—hindlimb paralysis; 4—tetraplegia; 5—moribund. Age- and sex-matched uninjected mice were used as controls (day 0).

### Primary Astrocyte Isolation

Neonatal astrocytes were purified from the brains of 1-day-old Sprague–Dawley rat pups and of RGC-32 KO and WT mouse pups as previously described (25). After removal of the meninges, the brains were minced and sequentially passed through nylon meshes. The resulting cell suspensions were plated onto 75-cm^2^ plates in DMEM/Ham’s F-12 medium containing 10% fetal bovine serum (FBS) for 2 weeks. Oligodendrocyte precursor cells and neurons were separated from the astrocyte monolayer by shaking overnight at 200 rpm on a rotary shaker. The non-adherent cell suspension was discarded, and adherent astrocytes were then maintained in DMEM/Ham’s F-12 medium containing 10% FBS. More than 97% of the cells isolated expressed the astrocyte marker GFAP. Astrocytes were serum-starved overnight prior to stimulation with 10 ng/ml TGF-β (Gibco, Gaithersburg, MD).

### Immunohistochemistry

Control (day 0) and mice with EAE were sacrificed on day 14 under terminal anesthesia and perfused transcardially with 4% paraformaldehyde in PBS. Spinal cords were harvested, fixed in formaldehyde, and then paraffin embedded. Paraffin sections (5 μm) were deparaffinized and rehydrated by serial washes in xylene and alcohol and then washed in PBS. Antigen retrieval was achieved by immersion of slides in citrate buffer (pH 6) and boiling at 95°C for 30 min, followed by cooling of the sections at room temperature (RT) for 20 min. The endogenous peroxidase was quenched with hydrogen peroxide (3% in PBS) for 10 min, and the non-specific binding was blocked with 2.5% normal horse serum (Vector Labs, Burlingame, CA) for 30 min. The sections were then incubated with primary antibody: monoclonal rabbit anti-GFAP (IgG; Cell Signaling Technology, Danvers, MA), monoclonal rabbit anti-vimentin (IgG; Cell Signaling Technology) or polyclonal rabbit anti-FABP7 (IgG; Proteintech, Rosemont, IL) overnight at 4°C. The next day, the sections were incubated with a labeled secondary antibody, horseradish peroxidase (HRP)-conjugated anti-rabbit IgG polymer reagent (Vector Laboratories, Burlingame, CA), for 30 min at RT. The colorimetric reactions were developed using a NovaRed Peroxidase Substrate kit (Vector Labs). The sections were subsequently washed in distilled water and counterstained with Harris hematoxylin (Sigma-Aldrich, St. Louis, MO). The sections were then dehydrated and mounted. Control sections were prepared by immunostaining without the primary antibody.

For double-staining immunohistochemistry, deparaffinization, rehydration, antigen retrieval, endogenous peroxidase quenching, and blocking were achieved as described above. Sections were incubated overnight at 4°C with either goat polyclonal anti-CTGF IgG (Santa Cruz Biotechnology, Dallas, TX) or rabbit polyclonal anti-Ki67 (NeoMarkers, Portsmouth, NH). The next day, sections previously incubated with rabbit anti-Ki67 antibody were incubated with unconjugated Fab fragments of goat anti-rabbit IgG (Jackson ImmunoResearch Laboratories, West Grove, PA) for 1 h at RT for species conversion and then incubated with HRP-conjugated anti-goat IgG (Vector Labs) for 30 min at RT. Sections previously incubated with goat anti-CTGF were directly incubated with HRP-conjugated anti-goat IgG. The colorimetric reaction was developed using NovaRed (Vector Labs). After color development, endogenous alkaline phosphatase was inhibited with Bloxall Blocking Solution (Vector Labs) for 10 min, and the slides were then incubated with rabbit monoclonal anti-GFAP (IgG; Cell Signaling Technology) overnight at 4°C. The next day, the sections were incubated with alkaline phosphatase-conjugated goat anti-rabbit IgG (Vector Labs) for 1 h at RT, and the colorimetric reaction was developed with a Vector Blue Alkaline Phosphatase Substrate Kit (Vector Labs).

### Quantification of Immunohistochemistry

For quantification of the GFAP immunostaining, we measured the chromogenic density by using the Fiji version of ImageJ. First, the RGB images were split into three channels by using the hematoxylin and DAB (H DAB) vector of the Color Deconvolution tool: a DAB image corresponding only to the peroxidase staining, the hematoxylin image corresponding only to the hematoxylin counterstaining, and the residual image with no staining. The DAB image was chosen for analysis. We defined the region of interest (ROI) as the white matter area of 10x-magnified spinal cord sections and then measured the mean intensity of the ROI. The raw values were converted to optical density (OD) values by applying the following formula: OD = log (max intensity/mean intensity), where max intensity was 255, corresponding to the white, non-stained areas (27).

For vimentin and FABP7 immunostaining and for CTGF/GFAP and Ki-67/GFAP double immunostaining, we manually counted the number of vimentin- and FABP7-positive cells that resembled astrocytes and radial glia, and the number of CTGF/GFAP- and Ki-67/GFAP-double-positive cells by using the Point Tool analysis of the ImageJ. We counted the total number of those cells present in the ROI corresponding to white matter areas of 10x- or 20x-magnified spinal cord sections. Quantification of single- and double-staining immunohistochemistry was performed in a blinded fashion.

### Immunocytochemistry

WT and RGC-32 KO mouse astrocytes were plated on Permanox^®^ Chamber Slide culture chambers (Nunc Inc, Rochester, NY) for 24 h, fixed in acetone for 10 min, and then air-dried. The sections were washed in PBS and then incubated with 0.3% hydrogen peroxide diluted in PBS for 10 min to quench the endogenous peroxidase. The slides were incubated with primary rabbit anti-GFAP antibody (Dako Agilent, Santa Clara, CA) overnight at 4°C and then with HRP-conjugated anti-rabbit IgG (Vector Labs) for 30 min. NovaRed (Vector Labs) was used as the chromogenic substrate.

### Transfection of Astrocytes

Primary rat astrocytes were transiently transfected with RGC-32 siRNA (siRGC-32) or control siRNA (siCTR) (Santa Cruz Biotechnology) by using Lipofectamine 3000 (Invitrogen, Carlsbad, CA): 75 pmol of siRNA and 7.5 μl of Lipofectamine 3000 were diluted separately in Opti-MEM I Reduced Serum Medium (Gibco, Waltham, MA), incubated for 5 min at RT, and then combined and incubated for 10 min at RT. The mixture was then added to the astrocytes, which had been plated 1 day earlier on 6-well plates or 25-cm^2^ flasks at a confluence of ~70% in antibiotic-free DMEM/Ham’s F-12 supplemented with 10% FBS, according to the manufacturer’s instructions. At 48 h after transfection, astrocytes were starved in serum-free DMEM/F-12 overnight and then treated with 10 ng/ml TGF-β or vehicle for the indicated periods of time.

### RNA Isolation and cDNA Synthesis

Total RNA from astrocytes was purified using an RNeasy Mini Kit (Qiagen, Germantown, MD) according to the manufacturer’s instructions. RNA (0.5 μg per sample) was mixed with reverse transcriptase buffer (Promega, Madison, WI), dNTPs (Promega), and random primers (Invitrogen). The RNA was denatured by incubation at 65°C for 5 min. M-MLV reverse transcriptase (Promega) was then added, and the reaction mixture was incubated at 37°C for 1 h. The reaction was terminated by incubation of the mixture at 95°C for 5 min.

### Quantitative Real-Time PCR

Forward and reverse primers for CTGF, IGF1, IGFBP2, IGFBP3, MMP2, MMP9, and PLAUR were provided by Integrated DNA Technologies (Coralville, IA) (**Supplementary Table**). 18S was used as an internal control. Real-Time PCR was performed according to the manufacturer’s protocol using FastStart Universal SYBR Green Master Mix (Roche, Indianapolis, IN) and a StepOnePlus Real-Time PCR System (Applied Biosystems, Carlsbad, CA). Quantification was performed by using the ΔΔCT method of relative quantification as previously described (28).

### Real-Time PCR Array

Astrocytes were stimulated with 10 ng/ml TGF-β for 24 h, and total RNA was used for a predesigned 96-well PrimePCR ECM Remodeling panel with 40 targets (Biorad, Hercules, CA), according to the manufacturer’s instructions.

### Nuclear/Cytoplasmic Extract Preparation

Nuclear and cytoplasmic extracts of rat astrocytes were prepared by using a NEPER kit (Thermo Scientific). After washing and trypsinization for detachment, cells were resuspended in cytoplasmic extraction reagent I (CER I) and incubated on ice. Cytoplasmic extraction reagent II (CER II) was then added to the samples, and the extracts were further incubated on ice. The extracts were centrifuged for 5 min, and the supernatant containing the cytoplasmic fraction was transferred to a new tube. The pellet containing nuclear fraction was incubated with nuclear extraction reagent (NER) and then placed on ice for 40 min, with vortexing every 10 min. The samples were then centrifuged for 10 min, and the supernatant containing purified nuclear extracts was transferred to a new tube. Samples of nuclear and cytoplasmic extracts were further processed by Western blotting.

### Western Blotting

Western blotting was performed as previously described (25). Cells were lysed in RIPA buffer containing protease and phosphatase inhibitors. Whole-cell lysates (total protein = 10–30 μg) were electrophoresed on 10% SDS-PAGE, followed by transfer to nitrocellulose membranes. Membranes were incubated with the following primary antibodies overnight at 4°C: rabbit monoclonal anti-GFAP IgG (Cell Signaling Technology), rabbit monoclonal anti-vimentin IgG (Cell Signaling Technology), goat polyclonal anti-CTGF IgG (Santa Cruz Biotechnology), rabbit polyclonal anti-IGFBP3 IgG (Proteintech), rabbit polyclonal anti-IGFBP6 IgG (Proteintech), rabbit polyclonal anti-MMP9 IgG (Proteintech), rabbit polyclonal anti-STAT3 IgG (Santa Cruz Biotechnology). Mouse monoclonal anti-β-tubulin IgG (Proteintech) and rabbit polyclonal anti-β-actin IgG (Rockland Immunochemicals, Limerick, PA) were used as internal controls for whole extracts and cytoplasmic fractions, respectively, whereas rabbit monoclonal anti-H3 IgG (Cell Signaling Technology) was used as internal control for nuclear fractions. Anti-rabbit or anti-mouse HRP-conjugated antibody (Santa Cruz Biotechnology) was used as secondary antibody, with a 1 h incubation at RT. Immune complexes were visualized by using enhanced chemiluminescence (Denville Scientific Inc., Metuchen, NJ, United States) and autoradiography. The radiographic band density was measured using UN-SCAN-IT software, version 7.1 (Silk Scientific, Orem, UT).

### Growth Factor Array

A RayBio^®^ C-Series Mouse Growth Factor Array (RayBiotech, Peachtree Corners, GA) was used to test the expression of most important growth factors regulated by RGC-32. Cultured mouse astrocytes from WT and RGC-32 KO mice were stimulated with 10 ng/ml of TGF-β for 24 h, and then the supernatants were incubated with the membrane antibody arrays overnight at 4°C. The array was processed according to manufacturer’s instructions.

### Statistical Analysis

Comparisons between multiple groups were performed using two-way ANOVA with the Holm-Sidak test for multiple comparisons. Comparisons between two groups were performed using an unpaired two-tailed *t*-test. P-values < 0.05 were considered significant. Statistical analysis was performed using GraphPad Prism software, version 7.

## Results

### Lack of RGC-32 Affects Astrocyte Density and Morphology in Spinal Cord During EAE

**Figure 1:**
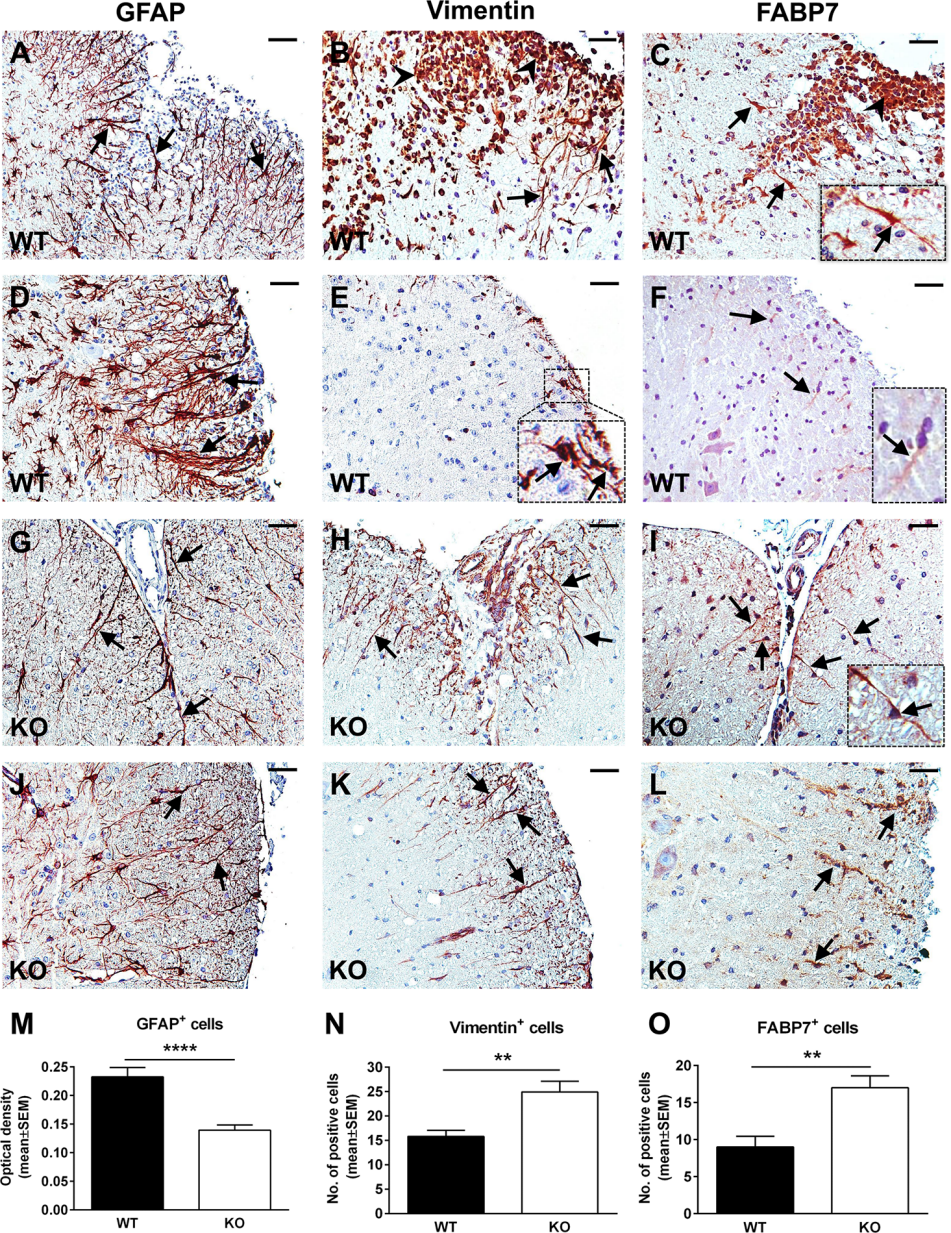
A lack of RGC-32 affects astrocyte density, process morphology, and intermediate filament protein expression in EAE spinal cords. Spinal cords from WT **(A–F)** and RGC-32 KO **(G–L)** mice were harvested at the peak of EAE (day 14) and stained with anti-GFAP **(A, D, G, J)**, anti-vimentin **(B, E, H, K)**, or anti-FABP7 antibody **(C, F, I, L)**, and then counterstained with Harris hematoxylin. GFAP-stained astrocytes from WT spinal cords show reactive changes in areas rich in inflammation (**A**, **D**, arrows), whereas RGC-32 KO spinal cords astrocytes have a bipolar morphology reminiscent of radial glia and astrocyte progenitors (**G**, **J**, arrows). Some of these reactive astrocytes are also positive around the inflammatory infiltrate for vimentin and FABP7 in WT spinal cords (**B**, **C**, arrows and insert), but they are scarce in areas outside of the inflammation (**E**, **F**, arrows and inserts). In contrast, vimentin- and FABP7-positive astrocytes with radial glia morphology are present throughout the spinal cords in KO mice (**H**, **I**, **K**, **L**, arrows). We noticed that the inflammatory infiltrate is also staining positive for vimentin and FABP7 (**B**, **C**, arrowheads). The blood vessels were also vimentin-positive **(H)**. Original magnification: x20. Scale bars: 40 µm. OD of the white matter GFAP-positive cells was determined from 10x-magnified spinal cord sections, and the mean OD per area was compared between WT and RGC-32 KO spinal cords as described in Materials and Methods. A significantly higher OD value for GAFP^+^ cells was seen in WT than in RGC-32 KO spinal cords **(M)**. Vimentin- and FABP7-positive cells with astrocyte and radial morphology were manually counted from white matter areas of 10x-magnified spinal cord sections, and the values were compared between WT and RGC-32 KO spinal cords. A significantly higher number of vimentin- and FABP7-positive cells were observed in RGC-32 KO spinal cords **(N, O)**. Results are expressed as mean ± SEM (**M**: n = 11 areas per 4 mice in WT; n = 14 areas per 5 mice in RGC-32 KO. **N**: n = 13 areas per 4 mice in WT; n = 17 areas per 4 mice in RGC-32 KO. **O**: n = 8 areas per 3 mice in WT; n = 10 areas per 4 mice in RGC-32 KO). ** = p<0.01; **** = p<0.0001.

The typical course of EAE is characterized by an accumulation of inflammatory infiltrate in the spinal cord during the acute phase, which usually peaks around day 14 (29). In parallel, reactive astrogliosis begins during the early stages of disease and reaches its maximum at the peak, strongly correlating with the presence of an inflammatory infiltrate and with the severity of the clinical course (15). RGC-32 KO mice with EAE show a statistically significant lower clinical score at the peak of the disease than WT mice (**Supplementary Figure 1**), in accordance to our previous studies which also demonstrated a decreased presence of inflammatory infiltrates in RGC-32 KO mice (23, 25). Therefore, we were interested in further exploring the reactive changes in astrocytes in RGC-32 KO mice. By using GFAP immunostaining, we analyzed the morphology, distribution, and density of spinal cord astrocytes from WT and RGC-32 KO mice with EAE at day 14. We found that astrocytes displayed a higher density of processes in WT spinal cords in areas rich in inflammatory infiltrate (**Figures 1A, D**) when compared to their RGC-32 KO counterparts (**Figures 1G, J**), which showed a drastic reduction in inflammatory infiltrate accumulation. Moreover, many WT astrocytes displayed morphological changes specific for reactive astrocytes, such as branching and hypertrophy (**Figure 1A**, arrows), together with process bundling (**Figure 1D**, arrows). In contrast, astrocytes from RGC-32 KO mice lacked reactive changes and showed less branching and thinner processes, and an overall bipolar morphology (**Figures 1G, J**, arrows) similar to the morphology of radial glia and immature astrocyte progenitors. When we measured the OD of the GFAP immunostaining in white matter, we found a statistically significant higher mean OD value per area in WT spinal cords than in RGC-32 KO spinal cords (p<0.0001; **Figure 1M**).

### EAE Spinal Cord Astrocytes With an Immature Morphology Have Higher Expression of Astrocyte Progenitor Markers in the Absence of RGC-32

Radial glia are neural stem cells that generate white matter fibrous astrocytes in the adult spinal cord (30). Some radial glia persists into adulthood and may serve as a source of reactive astrocytes during EAE through proliferation and hypertrophy (31, 32). We analyzed the expression and distribution of these cells by using vimentin and FABP7 as markers. Our results showed that in RGC-32 KO mice, vimentin- and FABP7-positive cells with a radial glial morphology were distributed throughout the spinal cord white matter, including in areas with no inflammation (**Figures 1H, K**, arrows for vimentin; **Figures 1I, L**, arrows and insert for FABP7), with their typical bipolar shape and long processes extending from the pial surface toward the grey matter (**Figures 1H, I**, arrows). In contrast, vimentin- and FABP7-positive astrocytes in WT mice showed branching and process hypertrophy and were distributed mostly around the inflammatory infiltrate (**Figure 1B**, arrows for vimentin; **Figure 1C**, arrows and insert for FABP7), with very few cells outside the area of inflammation (**Figure 1E**, arrows and insert for vimentin; **Figure 1F**, arrows and insert for FABP7), suggesting that they were more likely reactive astrocytes, since during reactivation, astrocytes can re-express these markers of immaturity (33). Manual counts of the number of vimentin- and FABP7-positive cells with astrocyte and radial morphology revealed a significantly higher number of these cells (p<0.01, **Figure 1N** for vimentin; p<0.01, **Figure 1O** for FABP7) in RGC-32 KO spinal cords than in WT spinal cords.

Taken together, our results show that in the absence of RGC-32, white matter spinal cord astrocytes are distributed with a lower density and display mostly an immature, radial glial-like phenotype that does not undergo the reactive changes typically seen at the peak of EAE, as was the case with WT mice.

### EAE Spinal Cord Astrocytes Have a Higher Proliferative Index in the Absence of RGC-32

**Figure 2:**
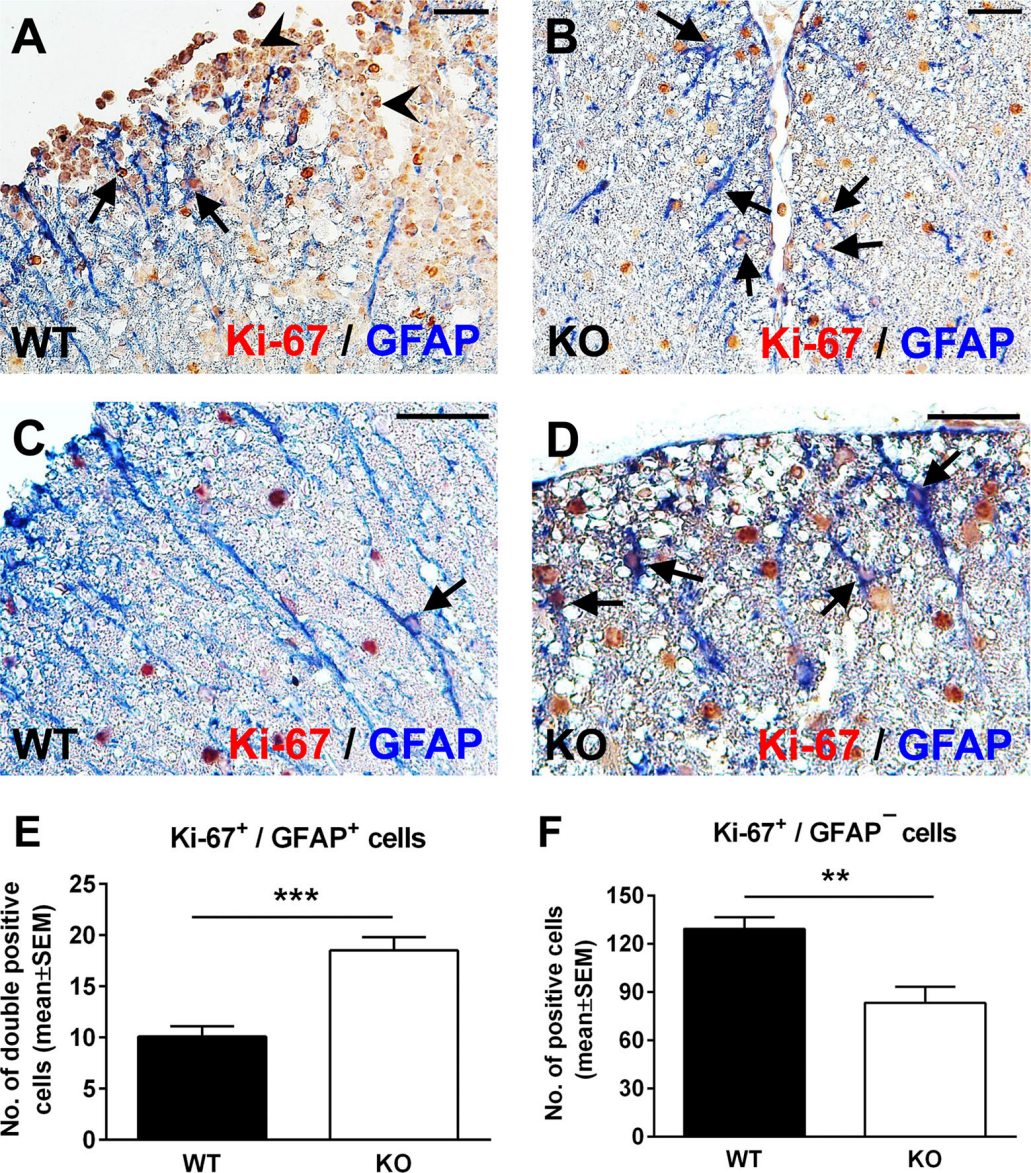
Lack of RGC-32 results in a higher proliferative index in spinal cord astrocytes during acute EAE. Spinal cords from WT **(A, C)** and RGC-32 KO mice **(B, D)** were harvested at the peak of EAE (day 14) and double-stained with anti-Ki-67 (red) and anti-GFAP (blue) antibody. In WT mice, Ki-67-positive astrocytes were mainly present around the inflammation (**A**, arrows) with few cells outside inflammatory infiltrate (**C**, arrow). In contrast, in RGC-32 KO mice, we noticed abundant Ki-67-positive astrocytes throughout the spinal cords (**B**, **D**, arrows). Original magnification: **(A, B)**: x20; **(C, D)**: x40. Scale bars **(A, B)**: 40 µm; **(C, D)**: 20 µm. Ki-67-positive/GFAP-positive and Ki-67-positive/GFAP-negative cells were manually counted in white matter areas from 10x-magnified spinal cord sections, and the values were compared for WT and RGC-32 KO spinal cords. We found a higher number of Ki-67/GFAP double-positive cells in the RGC-32 KO mice **(E)** and a lower number of Ki-67-positive/GFAP-negative cells in these mice than in the WT mice **(F)** because of the presence of inflammatory cells positive for Ki-67 (**A**, arrowheads). Results in **(E, F)** are expressed as mean ± SEM (n = 7 areas per 3 mice in WT: n = 6 areas per 3 mice in RGC-32 KO). ** = p<0.01; *** = p<0.001.

Spinal cord astrocytes are formed through a series of coordinated steps involving cell lineage specification, migration, proliferation, terminal differentiation, and maturation (34). Since RGC-32 has been found to participate in regulating cell cycle progression in a number of cell types (2, 3), we hypothesized that the decreased density of astrocytes in RGC-32 KO spinal cords and their immature phenotype might be related to a defect in their proliferation. We therefore analyzed the expression of the proliferative marker Ki-67 in astrocytes from our WT and RGC-32 KO EAE mice by using double staining for Ki-67 and GFAP. Surprisingly, Ki-67/GFAP-double-positive cells appeared to be abundant throughout the spinal cord white matter in RGC-32 KO mice, including areas without inflammatory infiltrate (**Figures 2B, D**, arrows); however, the overall number of Ki-67-positive cells was higher in WT spinal cords, mostly because of the presence of inflammatory infiltrates (**Figure 2A**, arrowheads). In WT spinal cords, Ki-67/GFAP-double-positive cells were present around the infiltrates (**Figure 2A**, arrows), with only a few cells outside the areas of inflammation (**Figure 2C**, arrow). When we quantified the total number of Ki-67/GFAP-double-positive cells and of Ki-67-positive/GFAP-negative cells, we found a significantly higher number of Ki-67/GFAP-double-positive cells in RGC-32 KO spinal cords (p<0.001; **Figure 2E**). As expected, the total number of Ki-67-positive/GFAP-negative cells were significantly higher in the WT spinal cords (p<0.01; **Figure 2F**).

Taken together, these results demonstrate that RGC-32 KO astrocytes, including radial glia, have a higher proliferative index than do the analogous cells in WT mice, and this difference might be explained by the fact that RGC-32 can inhibit the cell cycle in some types of cells, as we have already shown for CD4^+^ T-cells (26).

### The Lack of Reactive Changes in RGC-32 KO Astrocytes Reflects Their Intrinsic Inability to Differentiate Into a Mature Phenotype

**Figure 3:**
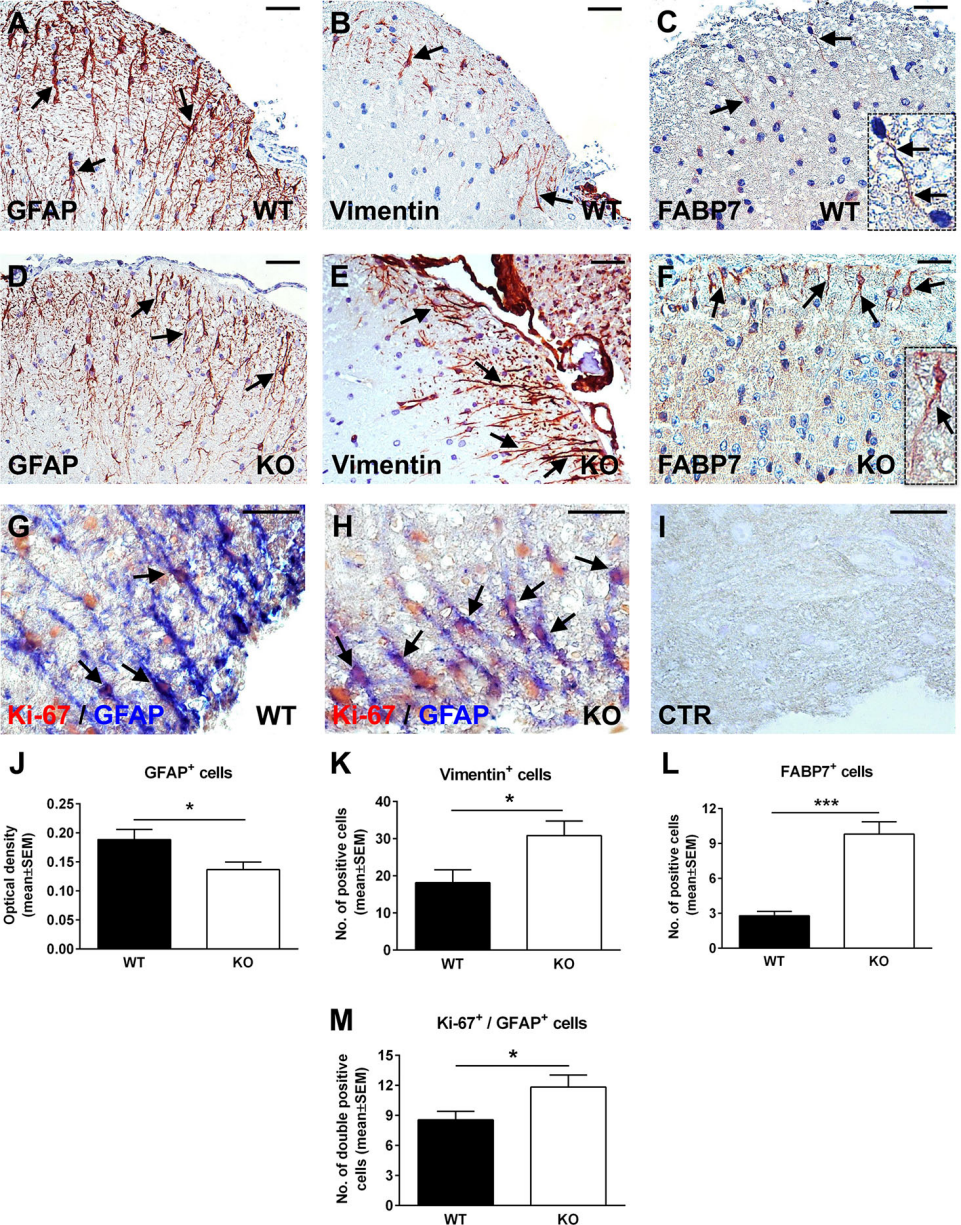
Lack of RGC-32 affects astrocyte density, morphology, proliferation index, and the number of vimentin and FABP7-positive radial glia in normal adult mouse spinal cords. Spinal cords were harvested from normal 8-week-old control WT **(A–C, G)** or RGC-32 KO mice **(D–F, H)**, and were stained with anti-GFAP **(A, D)**, anti-vimentin **(B, E)**, anti-FABP7 antibody **(C, F)**, and then counterstained with Harris hematoxylin or were double-stained with anti-Ki-67 (red) and anti-GFAP (blue) antibody **(G, H)**. GFAP immunostaining shows more densely arranged astrocytes with thicker processes in the WT mice (**A**, arrows) than in the RGC-32 KO mice (**D**, arrows). RGC-32 KO mice show a higher density of vimentin-positive radial glia (**E**, arrows) than do WT mice (**B**, arrows). FABP7-positive radial glia cells are present in the RGC-32 KO mice (**F**, arrows and insert) but are barely detectable in the WT mice (**C**, arrows and insert). Ki-67/GFAP double staining shows that RGC-32 KO mice display a higher number of astrocytes with Ki-67-positive nuclei (**H**, arrows) than do WT mice (**G**, arrows). Controls (CTR) for the immunoperoxidase and alkaline phosphatase reactions were negative **(I)**. Original magnification: **(A–F)** x20; **(G–I)** x40. Scale bars: **(A–F)** 40 µm; **(G–I)** 20 µm. The GFAP immunostaining OD and the number of vimentin- and FABP7-positive radial glia were assessed as previously described in **Figures 1** and **2**. We found a significantly higher OD values for GFAP in WT mice **(J)** and a significantly higher number of vimentin- and FABP7-positive radial glia in RGC-32 KO mice **(K, L)**. Ki-67-positive/GFAP-positive cells were manually counted in white matter areas corresponding to x20-magnified spinal cord sections. We found a significantly higher number of Ki-67/GFAP double-positive cells in the RGC-32 KO mice **(M)**. Results in **(J–M)** are expressed as mean ± SEM (**J**: n = 6 areas per 2 mice in WT; n = 5 areas per 2 mice in RGC-32 KO. **K**: n = 6 areas per 2 mice in both groups. **L**: n = 5 areas per 2 mice in both groups. **M**: n = 9 areas per 2 mice in WT: n = 6 areas per 2 mice in RGC-32 KO). * = p<0.05; *** = p<0.001.

Since RGC-32 KO mice have less inflammatory infiltrate in their spinal cords at the peak of the EAE course (23), one possibility is that the lack of reactive changes that we observed might have resulted from an insufficient inflammatory stimulus. However, our previous work has shown that siRNA-mediated silencing of RGC-32 reduces the TGF-β-induced expression of reactive astrocyte markers and synthesis of ECM components *in vitro* (25), pointing to an important contribution by RGC-32 in directly modulating astrocyte reactivity. To gain more insight into the possibility that RGC-32 deletion affects astrocytes differentiation *per se*, we analyzed the expression of GFAP, vimentin, and FABP7 in spinal cords from normal 2-month-old WT and RGC-32 KO mice. By using GFAP immunohistochemistry, we found a higher density of white matter astrocytes in WT spinal cords, with thicker and more highly branched processes (**Figure 3A**, arrows). In contrast, astrocytes from RGC-32 KO mice were slightly thinner, less densely organized, and less highly branched (**Figure 3D**, arrows). The mean value of OD per area of the GFAP immunostaining was significantly higher in the WT group, as measured by the method described above (p<0.05; **Figure 3J**). In a head-to-head comparison of GFAP immunostaining OD values between control mice at day 0 and mice with EAE at day 14, we found that WT mice with EAE had a significantly higher OD value at day 14 as compared to day 0 (0.189 ± 0.018 at day 0, 0.233 ± 0.016 at day 14; p=0.04). In contrast, we didn’t see any significant increase of OD at day 14 as compared to day 0 in RGC-32 KO mice (0.137 ± 0.013 at day 0, 0.140 ± 0.009 at day 14; p=0.9), suggesting that reactive changes occurred in WT astrocytes but not in RGC-32 KO astrocytes.

When we analyzed vimentin expression, we observed the presence of radially oriented vimentin-positive cells in both groups. However, RGC-32 KO mice showed a higher density of vimentin-positive cells (**Figure 3E**, arrows) than did WT mice (**Figure 3B**, arrows). There was also a significantly higher number of vimentin-positive cells per area in RGC-32 KO mice when compared to WT mice (p<0.05, **Figure 3K**). In a similar manner, FABP7-positive cells with a radial morphology were more numerous and showed thicker processes in the RGC-32 KO spinal cords (**Figure 3F**, arrows and insert) than in the WT spinal cords, in which such glia were only barely detected and had very thin processes (**Figure 3C**, arrows and insert). Overall, the number of FABP7-positive cells per area was significantly higher in RGC-32 KO spinal cords (p<0.001; **Figure 3L**).

**Figure 4:**
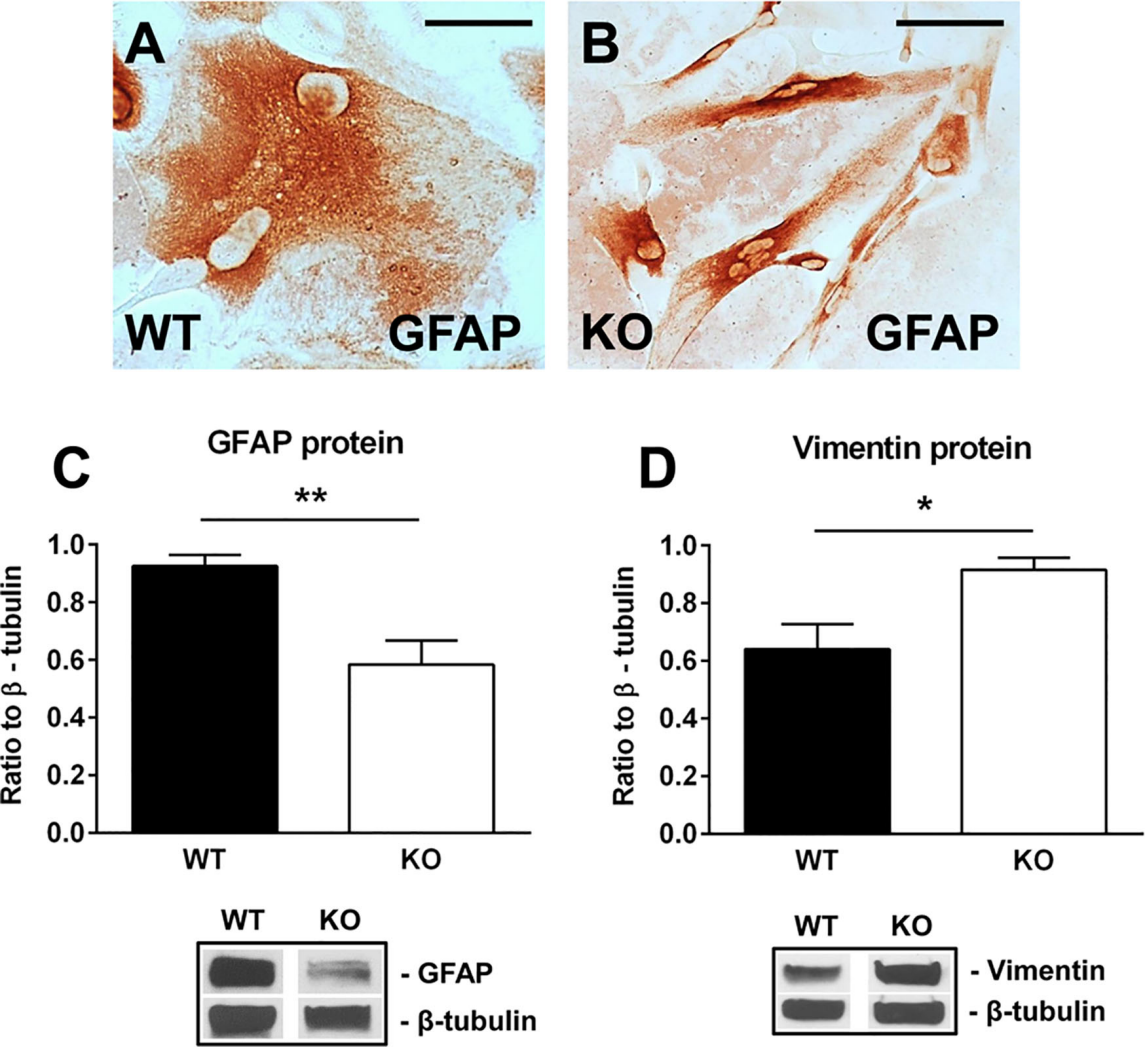
Effect of RGC-32 deletion on GFAP and vimentin expression *in vitro* in mouse astrocytes. WT and RGC-32 KO mouse astrocytes were isolated from the brains of 2-day-old pups and grown in DMEM/F-12 medium supplemented with 10% fetal bovine serum for 2 weeks, plated on a slide culture chamber, and then stained with anti-GFAP antibody. WT astrocytes show a mature morphology **(A)**, whereas RGC-32 KO astrocytes have a radial glia like morphology **(B)**. Original magnification: x40. Scale bars: 10 µm. The expression of GFAP and vimentin was analyzed by Western blotting and normalized to β-tubulin. Significantly higher levels of GFAP protein are seen in WT astrocytes than in RGC-32 KO astrocytes **(C)**, whereas vimentin levels are higher in KO astrocytes **(D)**. Results in **(C, D)** are expressed as mean ± SEM (n=3), and a representative blot is shown for each protein. * = p<0.05; ** = p<0.01.

We then purified neonatal astrocytes from WT and RGC-32 KO mouse pups and cultured them in differentiation medium. After 2 weeks, the astrocytes were analyzed for the expression of GFAP and vimentin by Western blotting and immunocytochemistry. GFAP immunostaining showed that WT astrocytes displayed body hypertrophy with a mature, differentiated phenotype (**Figure 4A**), whereas in the case of RGC-32 KO mice, many of the astrocytes showed an elongated, bipolar shape closely resembling that of radial glial cells (**Figure 4B**). Moreover, Western blot analysis demonstrated significantly higher levels of GFAP protein in WT astrocytes than in RGC-32 KO astrocytes (**Figure 4C**), whereas vimentin levels were higher in RGC-32 KO astrocytes (**Figure 4D**), paralleling the differences seen *in vivo* in the spinal cords.

These results suggest that RGC-32 is necessary for the completion of astrocyte differentiation and that in the absence of RGC-32, astrocytes are intrinsically unable to fully differentiate into a mature phenotype and to undergo reactive changes during EAE, instead retaining a radial glial-like phenotype.

### RGC-32 KO Astrocytes From Normal Adult Spinal Cords Retain Their Higher Proliferative Index

Next, we were interested to see whether astrocytes from normal adult naïve mice that lack RGC-32 also have a higher proliferative index. Using double-staining immunohistochemistry for Ki-67 and GFAP, we found that, as in the EAE spinal cords, RGC-32 KO astrocytes expressed a higher number of Ki-67-positive nuclei (**Figure 3H**, arrows) than did WT astrocytes (**Figure 3G**, arrows). In addition, the total number of Ki-67/GFAP-double-positive cells was significantly higher in the RGC-32 KO group (**Figure 3M**), consistent with their increased proliferative index.

### RGC-32 Regulates a Complex Network of Genes Associated With Cell Growth, Cell Motility, Extracellular Matrix Remodeling, and Signal Transduction in Astrocytes

The above-mentioned findings raised important questions about how RGC-32 modulates astrocyte differentiation and their ability to respond to inflammatory infiltrates during EAE, prompting us to further investigate the possible mechanistic pathways involved.

**Figure 5:**
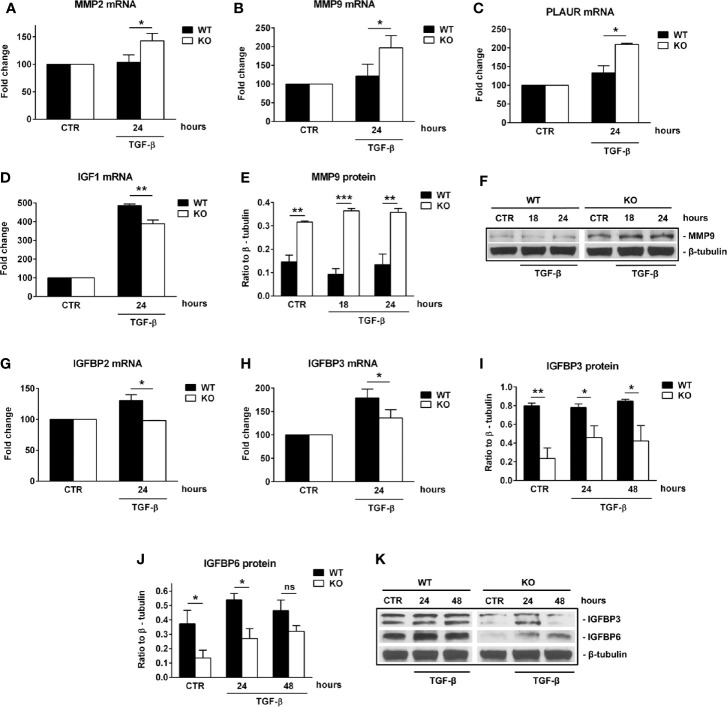
RGC-32 regulates the expression of MMP2, MMP9, PLAUR, IGF1 and IGFBPs *in vitro.* WT and RGC-32 KO mouse astrocytes were treated with 10 ng/ml TGF-β for the indicated periods of time. The expression of MMP2, MMP9, PLAUR, IGF1, IGFBP2 and IGFBP3 mRNAs was assessed by Real-Time PCR. The expression of MMP9, IGFBP3 and IGFBP6 proteins was assessed by Western blotting. MMP2 **(A)**, MMP9 **(B)**, and PLAUR mRNAs **(C)** were found to be significantly induced at 24 h of stimulation in RGC-32 KO astrocytes. MMP9 protein was also found to be significantly higher both under basal conditions and after 18 and 24 h of stimulation in RGC-32 KO mice when comparted to WT mice **(E, F)**. In contrast, IGF1 **(D)**, IGFBP2 **(G)** and IGFBP3 mRNAs **(H)** were significantly higher at 24 h of stimulation in WT astrocytes than in RGC-32 KO astrocytes. Also, the levels of IGFBP3 protein were higher under basal conditions and at 24 and 48 h of stimulation in the WT astrocytes than in the RGC-32 KO astrocytes **(I, K)**. IGFBP6 protein expression was significantly higher in WT astrocytes under basal conditions and after 24 h of stimulation **(J, K)**. The expression of the mRNA at the beginning of the experiment (CTR) was considered to be 100, and the results are shown as -fold change **(A–D, G–H)**. The protein level is expressed as a ratio to β-tubulin, and representative blots are shown **(F, K)**. Data are expressed as mean ± SEM (n=3). * = p<0.05; ** = p<0.01; *** = p<0.001; ns = not statistically significant.

First, we performed a qPCR array in search of genes that may be differentially regulated by RGC-32. We stimulated WT and RGC-32 KO mouse astrocytes with TGF-β, a potent cytokine known to induce both astrocyte differentiation and reactivity changes (35, 36). Differential analysis showed that most of the genes whose expression was affected by the lack of RGC-32 were related to cell growth and motility, extracellular matrix remodeling, and signal transduction (**Table 1**). ECM components such as COL1A1 were highly induced in WT astrocytes when compared to RGC-32 KO astrocytes, reflecting our previous results (25), as were genes involved in cell motility, such as CD44 and Ezrin (EZR). We also found differential regulation of genes that encode enzymes involved in ECM remodeling, such as matrix metalloproteinases (MMP) 3, 9, 13, 14; the plasminogen activator, urokinase receptor (PLAUR); and tissue inhibitor of metalloproteinases 1 (TIMP1) (**Table 1**). Subsequent Real-Time PCR and Western blot analysis confirmed the differential expression of MMP9 and PLAUR, with higher expression of MMP9 mRNA (**Figure 5B**) and protein (**Figures 5E, F**) and PLAUR mRNA (**Figure 5C**) in RGC-32 KO astrocytes after TGF-β stimulation. Another member of the MMP family, namely MMP2, was also found to have a higher expression in RGC-32 KO astrocytes at the mRNA level (**Figure 5A**). It could be that the decreased expression of MMP2 and MMP9 in WT astrocytes during TGF-β stimulation contributes to glial scar preservation by preventing the degradation of ECM, in line with the pro-gliotic effects of RGC-32 (25). As an alternative interpretation, the increased expression of MMP2 and MMP9 in RGC-32 KO astrocytes could reflect a greater capacity for ECM degradation and cell migration as part of a progenitor phenotype.

**Table 1:** Real Time PCR array.

Gene name	Genbank accession	WT fold change	KO fold change	WT/KO ratio
Insulin-like growth factor 1 receptor (IGF1R)	NT_187035.1	52.35	2.46	21.28
Collagen type I alpha 1 (COL1A1)	NT_096135.6	60.02	4.33	13.86
Ezrin (EZR)	NT_039636.8	36.78	2.78	13.23
Insulin-like growth factor 1 (IGF1)	NT_039500.8	17.88	3.57	5
Matrix metallopeptidase 13 (MMP13)	NT_039471.8	0.31	0.09	3.4
Epidermal growth factor receptor (EGFR)	NT_039515.7	2.84	0.98	2.9
CD44 antigen	NT_039207.8	5.87	2.26	2.6
Matrix metallopeptidase 3 (MMP3)	NT_039471.8	1.49	0.67	2.22
Kallikrein 1-related peptidase b16 (KLK1B16)	NT_187035.1	2.38	1.17	2.03
Serine (or cysteine) peptidase inhibitor, clade E, member 1 (SERPINE1)	NT_039314.8	12.43	18.62	0.66
Matrix metallopeptidase 14 (MMP14)	NT_039606.8	1.72	2.79	0.61
Tissue inhibitor of metalloproteinase 1 (TIMP1)	NT_039700.8	3.75	6.89	0.54
Plasminogen activator, urokinase receptor (PLAUR)	NT_187034.1	0.81	2.92	0.27
Matrix metallopeptidase 9 (MMP9)	NT_039207.8	0.44	1.69	0.26

WT and RGC-32 KO mouse astrocytes were stimulated with TGF-β1 (10 ng/ml) for 24 hours and total RNA was purified, reverse transcribed and subjected to a PrimePCR ECM remodeling array, according to manufacturer’s instructions. Each transcript was normalized to GAPDH. Quantification was performed by using the ΔΔCT method of relative quantification. The values are shown as fold change over unstimulated, which was considered 1. The results show the ratios with at least 1.5-fold difference between WT and KO.

Moreover, our array data showed that RGC-32 regulates the expression of a number of growth factors and their receptors, such as Insulin-like growth factor 1 (IGF1), IGF1 receptor (IGF1R), and epidermal growth factor receptor (EGFR), since their levels were higher in WT astrocytes (**Table 1**). Using Real-Time PCR, we were able to confirm the differential expression of IGF1: IGF1 mRNA levels were higher in WT astrocytes than in KO astrocytes after TGF-β stimulation (**Figure 5D**).

### RGC-32 Modulates the Secretion of Growth Factors and Their Regulatory Proteins in Astrocytes

Growth factors play an important role in astrocyte differentiation and proliferation, as well as in sustaining both normal astrocyte function and chronic astrogliosis (37–39). We therefore explored in further detail the relationship between RGC-32 and growth factor expression by using a mouse growth factor antibody array and the supernatants of WT and RGC-32 KO mouse astrocytes stimulated with TGF-β. We found that the levels of secreted growth factors such as vascular endothelial growth factor (VEGF), placental-derived growth factor (PlGF), platelet-derived growth factor AA (PDGF-AA), and hepatocyte growth factor (HGF), were higher in WT astrocytes than in RGC-32 KO astrocytes (**Table 2**). In contrast, isoform D of VEGF (VEGF-D) and the VEGF receptor 2 (VEGFR2) were increased in RGC-32 KO astrocytes, and their levels were almost non-detectable in WT astrocytes (**Table 2**), suggesting a complex regulation of the VEGF signaling pathway in astrocytes. Interestingly, expression of several members of the insulin-like growth factor binding protein family (IGFBP), such as IGFBP2, IGFBP5, and IGFBP6, was also higher in WT than in RGC-32 KO astrocytes (**Table 2**).

**Table 2:** Mouse growth factor antibody array.

Protein name	Genbank accession	WT fold change	KO fold change	WT/KO ratio
Hepatocyte growth factor (HGF)	AAI19229.1	1.3	0.57	2.28
Insulin-like growth factor binding protein 5 (IGFBP5)	AAH57447.1	1.68	0.75	2.24
Platelet-derived growth factor-AA (PDGF-AA)	NP_032834.1	2.26	1.2	1.88
Insulin-like growth factor binding protein 6 (IGFBP6)	EDL04005.1	1.46	0.87	1.67
Vascular endothelial growth factor A (VEGF-A)	NP_001020421.2	3.12	1.87	1.66
Placental growth factor (PlGF)	AAH16567.1	1.94	1.2	1.61
Insulin-like growth factor binding protein 2 (IGFBP2)	AAH54473.1	1.7	1.13	1.5
Vascular endothelial growth factor receptor 2 (VEGFR2)	AAH20530.1	1.09	1.77	0.61
Colony stimulating factor 1 (macrophage) (M-CSF)	AAH66205.1	0.71	1.25	0.56
Interleukin 2 (IL-2)	CAA25909.1	1.02	1.9	0.53
Vascular endothelial growth factor D (VEGF-D)	BAA14002.1	0.81	2.01	0.4

WT and RGC-32 KO mouse astrocytes were stimulated with TGF-β1 (10 ng/ml) for 24 hours. The conditioned media were collected and processed according to manufacturer’s instructions. The signals were quantified by densitometry and each protein was normalized to a standardized positive control provided in the array. The protein/positive control ratio after stimulation is expressed as fold change over unstimulated ratio, which was considered 1. The results show the ratios with at least 1.5-fold difference between WT and KO.

We also found that IGFBP2 and IGFBP3 mRNA levels were significantly higher in WT than RGC-32 KO astrocytes at 24 h of TGF-β stimulation (**Figures 5G, H**). Furthermore, the protein level of IGFBP3 was significantly lower in RGC-32 KO mouse astrocytes before stimulation and remained low after 24 h and 48 h of TGF-β stimulation when compared to WT mouse astrocytes (**Figures 5I, K**). IGFBP6 protein levels were significantly higher in WT astrocytes under basal conditions and after 24 h of TGF-β stimulation (**Figures 5J, K**). In a similar way, we found that the protein expression of IGFBP2 and IGFBP3 was significantly reduced after RGC-32 silencing by transfecting rat astrocytes with siRGC-32 as compared to siCTR at 24 h of TGF-β stimulation (**Supplementary Figure 2**).

Taken together, our findings suggest that RGC-32 is important in modulating the secretion of a plethora of growth factors and their regulatory proteins and that IGFBP2, IGFBP3, and IGFBP6 are the three members of the IGFBP family which expression was differentially regulated by RGC-32.

### RGC-32 Regulates the Expression of CTGF *In Vitro* in Neonatal Astrocytes and *In Vivo* During EAE

**Figure 6:**
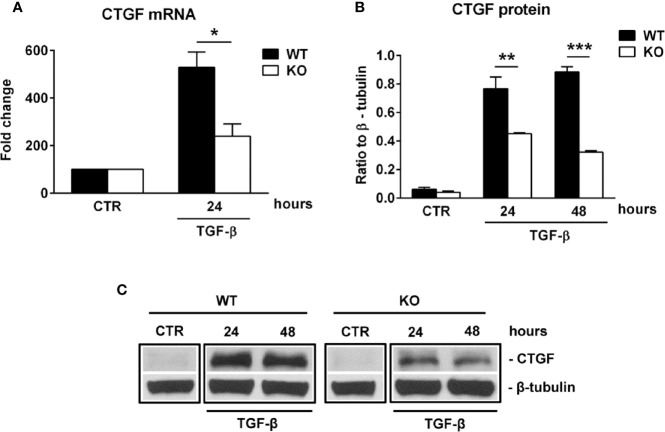
RGC-32 regulates the expression of CTGF *in vitro.* WT and RGC-32 KO mouse astrocytes were treated with 10 ng/ml TGF-β for the indicated periods of time and then examined for the expression of CTGF mRNA and protein by Real-Time PCR and Western blotting, respectively. CTGF mRNA expression was significantly higher at 24 h of stimulation in WT astrocytes **(A)**, and CTGF protein levels were higher at 24 h and 48 h of stimulation in WT astrocytes than in RGC-32 KO astrocytes **(B, C)**. The expression of the mRNA at the beginning of the experiment (CTR) was considered to be 100, and the results are shown as -fold change **(A)**. The protein level is expressed as a ratio to β-tubulin **(B)**, and a representative blot is shown in **(C)** Data are expressed as mean ± SEM (n=3). * = p<0.05; ** = p<0.01; *** = P<0.001.

Connective tissue growth factor (CTGF) is a member of the CCN family of growth factors, related to the IGFBP family. It has been shown to be important in astrocyte differentiation (40) and astrocyte-mediated inflammation, being able to activate astrocytes in an autocrine manner (41). Since our results suggest impaired differentiation and reactivity of RGC-32 KO astrocytes during EAE, we hypothesized that RGC-32 might exert its effects on astrocytes through CTGF. Indeed, cultured astrocytes stimulated with TGF-β showed a significantly higher level of CTGF mRNA in the WT group at 24 h of stimulation than did RGC-32 KO astrocytes (**Figure 6A**). At the protein level, CTGF expression was not detected in unstimulated cells but was significantly increased in the WT astrocytes at 24 h and 48 h of stimulation when compared to the RGC-32 KO astrocytes (**Figures 6B, C**).

**Figure 7:**
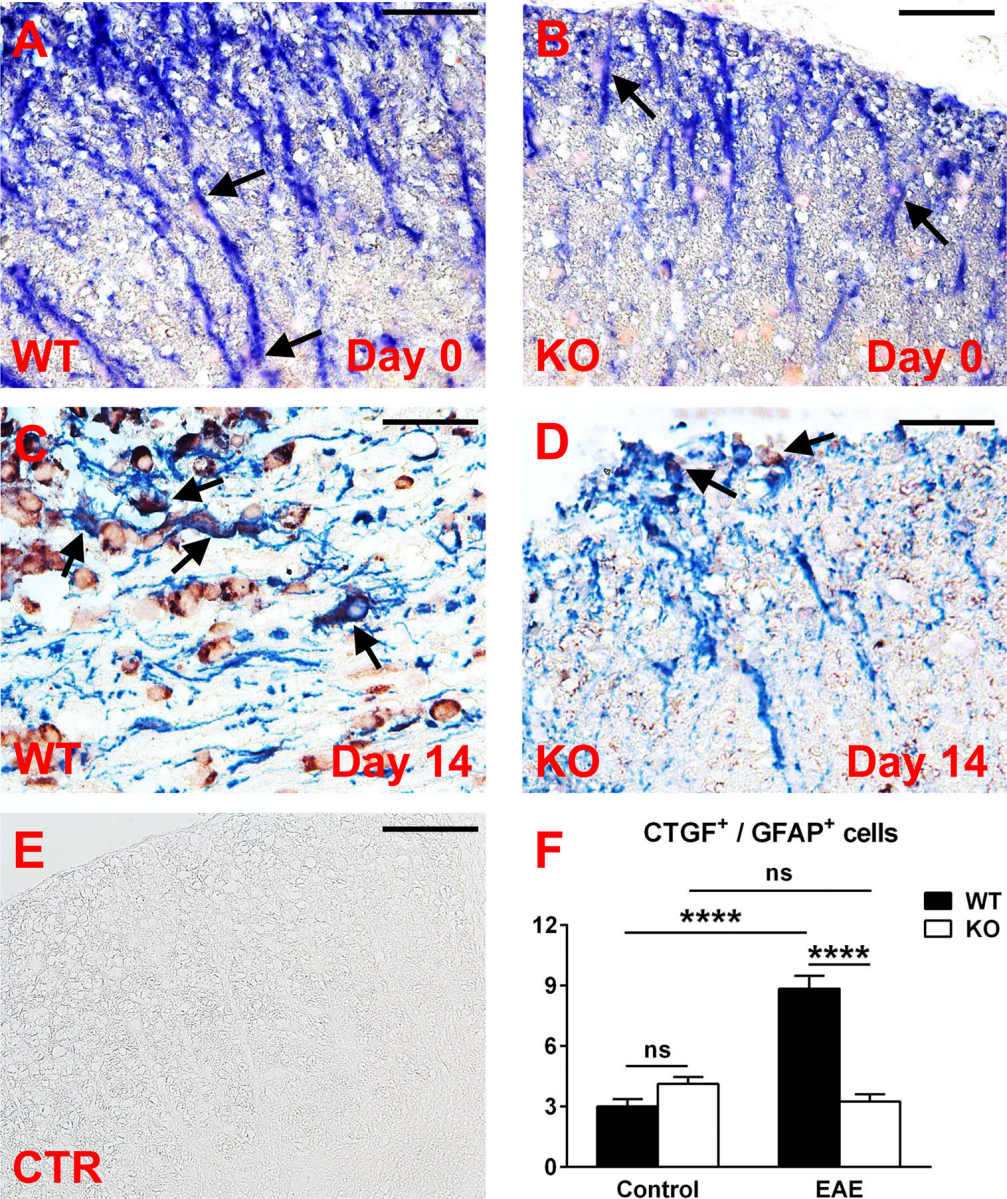
A lack of RGC-32 affects the number of CTGF-positive astrocytes in spinal cords from EAE mice but not in control mice. Spinal cords were harvested from control mice (day 0) and from mice with EAE at day 14 and double-stained with anti-CTGF (red) and anti-GFAP (blue) antibody. At day 0, CTGF^+^ astrocytes are barely detected in both groups (**A**, **B**, arrows), but during EAE they become abundant in WT mice (**C**, arrows), as compared to RGC-32 KO mice, where CTGF co-localization remains at a low level (**D**, arrows). Controls (CTR) for the immunoperoxidase and alkaline phosphatase reactions were negative **(E)**. Original magnification: x40. Scale bars: 20 µm. CTGF/GFAP-double-positive cells were manually counted in white matter areas corresponding to 20x-magnified spinal cord sections, and the mean value per area was compared between WT and RGC-32 KO mice, and between day 0 and day 14. No difference was detected between WT and RGC-32 KO mice at day 0. A significantly higher number of astrocytes with CTGF co-localization was observed in WT mice than in RGC-32 KO mice at day 14 **(F)**. Only WT mice showed a significant increase in CTGF^+^ astrocytes at day 14 as compared to day 0 **(F)**. Results in **(F)** are expressed as mean ± SEM (Day 0: n = 6 areas per 2 mice in WT; n = 8 areas per 2 mice in RGC-32 KO. Day 14: n = 7 areas per 2 mice in WT; n = 8 areas per 2 mice in RGC-32 KO). **** = p<0.0001; ns = not statistically significant.

We then analyzed the expression of CTGF in spinal cord astrocytes from both control and EAE mice. Double-staining immunohistochemistry for GFAP and CTGF showed that CTGF staining was barely detected in both WT and RGC-32 KO astrocytes at day 0 (**Figures 7A, B**, arrows). On the other hand, at day 14, CTGF staining was more intense in the GFAP-positive astrocytes from WT spinal cords (**Figure 7C**, arrows) than RGC-32 KO astrocytes, which showed very little CTGF co-localization (**Figure 7D**, arrows). When the number of CTGF/GFAP double-positive cells was counted and compared between WT and RGC-32 KO mice, we didn’t find any significant difference at day 0, but we found a significantly higher number of CTGF^+^ astrocytes in the WT spinal cords (**Figure 7F**) at day 14 as compared to RGC-32 KO mice. When we compared the number of CTGF^+^ astrocytes between day 0 and day 14, we observed a significant increase in the WT but not in the RGC-32 KO mice (**Figure 7F**).

In conclusion, our results clearly show that in the absence of RGC-32, CTGF levels remained lower in cultured astrocytes stimulated with TGF-β and in spinal cord astrocytes at the peak of EAE.

### RGC-32 Regulates the Nuclear Translocation of STAT3

STAT3 is a transcription factor with a critical role in driving astrocytogenesis from radial glia during the gliogenic switch (34) and in modulating glial scar formation as a response to spinal cord injury (21). Upon activation, STAT3 is translocated to the nucleus, where it exerts its transcriptional activity. We were interested to see whether RGC-32 influences the nuclear translocation of STAT3. For this purpose, we transfected rat astrocytes with siRNA against RGC-32 to abolish its expression, then stimulated the astrocytes with TGF-β and separated the nuclear and cytoplasmic fractions. Our results show that TGF-β stimulation significantly increased STAT3 nuclear translocation in siCTR-treated astrocytes (**Supplementary Figures 3A, B**), and this increase was significantly reduced after siRNA-mediated RGC-32 silencing (**Supplementary Figures 3A, B**), suggesting that RGC-32 is indeed involved in the nuclear translocation of STAT3 in astrocytes.

## Discussion

In this study, we have shown for the first time that spinal cord astrocytes from mice that lack RGC-32 exhibit characteristics of immaturity and an absence of reactive changes at the peak of EAE disease course, with a bipolar, elongated morphology similar to that of radial glia and astrocytic progenitors. In contrast, astrocytes from WT mice show the classical features of astrocytic reactivity, such as process branching and hypertrophy. Our immunohistochemical staining analysis demonstrated a difference in GFAP immunostaining, with levels being significantly lower in RGC-32 KO mice than WT mice.

**Figure 8:**
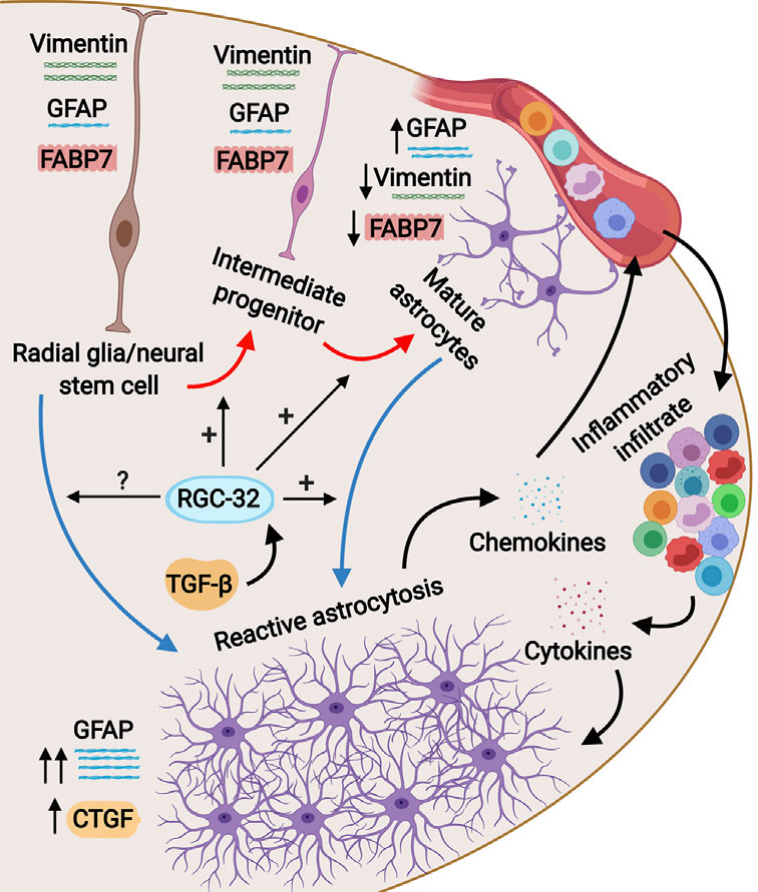
Schematic representation of the role of RGC-32 in astrocyte differentiation during EAE. Spinal cord astrocytes are generated from radial glia through a complex process involving cell lineage specification, migration, proliferation, terminal differentiation, and maturation. The presence of inflammatory infiltrate at the peak of EAE triggers astrocytes to become reactive and to upregulate their GFAP expression together with upregulating CTGF. Our results show that in the absence of RGC-32, astrocytes retain an elongated, bipolar shape and their elevated expression of progenitor markers such as vimentin and FABP7, but they are unable to undergo the morphological changes specific for reactive astrocytes and to upregulate their GFAP expression. It is currently not known whether RGC-32 also participates in adult radial glia-induced astrogliosis; these cells are an important source for reactive astrocytes in EAE.

Studies have shown that the reactive astrocytes in the white matter of mice with EAE are mostly derived from the hypertrophy and proliferation of a pre-existing population of mature fibrous astrocytes and adult radial glia (31, 42, 43). These reactive astrocytes exhibit hypertrophy of their cell bodies and processes, increase their GFAP expression, and can re-express progenitor cells markers such as vimentin, nestin, and FABP7, also known as brain lipid binding protein (BLBP) (42, 44). We found vimentin and FABP7 expression in both WT mice and RGC-32 KO mice with EAE, but the distribution, morphology, and number of astrocytes positive for these markers differed significantly between the two groups. In the WT mice, vimentin- and FABP7-positive astrocytes were distributed around the inflammatory infiltrate and showed morphological changes specific for reactive astrocytes, whereas in RGC-32 KO mice we found a higher number of vimentin- and FABP7-positive cells spread across the entire spinal cord section, with an elongated bipolar shape reminiscent of a radial glia morphology, suggesting that in the absence of RGC-32, most of these cells are in fact astrocyte progenitors and not vimentin- or FABP7-re-expressing reactive astrocytes. Interestingly, we noticed that the inflammatory cells stained positive for vimentin and FABP7 (**Figures 1B, C**, arrowheads), and therefore we excluded these cells from our analysis. We also found that blood vessels expressed vimentin (**Figure 1H**). This is not an unusual finding, since others have also noted the presence of vimentin in inflammatory cells and blood vessels (45, 46). Importantly, we also found a decreased density of astrocytes in the spinal cords of normal adult mice that lacked RGC-32, as well as an increased number of vimentin and FABP7-positive radial glia. GFAP immunostaining showed slight differences in the morphology of the astrocytes between normal WT and RGC-32 KO spinal cords, with thinner and less highly branched processes in the RGC-32 KO group. Our *in vitro* experiments showed that neonatal RGC-32 KO astrocytes had a higher expression of vimentin and a lower expression of GFAP under basal conditions, suggesting that RGC-32 regulates the expression of these intermediate filament proteins in neonatal astrocytes as well. Thus, based on these findings, we speculate that RGC-32 is essential for astrocyte differentiation and for the ability of astrocytes to undergo reactive changes during EAE (**Figure 8**).

A subset of radial glia persists throughout adulthood, and these cells are termed adult radial glia (32). In the brain, these cells contribute to adult neurogenesis (47), and in the spinal cord they are important reservoirs for reactive astrocytes after injury, providing new astrocytes through proliferation and hypertrophy (31, 32). We found a higher number of GFAP^+^ cells that were also positive for the proliferation marker Ki-67 in RGC-32 KO spinal cords from both naïve and EAE mice than in WT mice. RGC-32 also plays a role in cell cycle entry and proliferation of cells such as smooth muscle cells (2) and endothelial cells (3) and inhibits cells cycle activation in CD4^+^ T cells (26). Our data suggest that in the absence of RGC-32, these elongated, bipolar-shaped cells reminiscent of radial glia can proliferate, but they are unable to proceed to terminal differentiation.

Our results show that siRNA-mediated inhibition of RGC-32 expression in cultured rat astrocytes results in impaired nuclear translocation of STAT3. Since STAT3 is involved in driving the gliogenic switch, a set of transcriptional changes that trigger the activation of promoters of genes specific for astrocytic fate determination during astrocytogenesis (34, 48), these findings suggest that RGC-32 may affect astrocyte differentiation during this very early event. However, it is not known whether RGC-32 directly interacts with the STAT3 complex. Since we have previously demonstrated a physical interaction in astrocytes between RGC-32 and SMAD3 (25), it is possible that RGC-32 also interacts with STAT3 during the gliogenic switch.

We also found that many differentially regulated genes by RGC-32 after TGF-β stimulation belong to the IGF/IGFBP family of growth factors. The IGFs acting on IGF1R play critical roles in brain development and homeostasis. They regulate neuronal stem cell proliferation and differentiation, cell survival, synapse formation, and glucose and electrolyte balance (49). Astrocyte-derived IGF1 has been shown to have neuroprotective effects in stroke and brain ischemia (50, 51), as well as to protect against amyloid β-induced synapse and neuronal loss in Alzheimer’s disease (52). The bioavailability and function of IGFs are regulated by the IGFBPs, a family of six proteins (IGFBP1-6) with significant sequence homology (53). IGFBPs bind to IGFs and increase their circulatory half-life, thus decreasing their availability for receptor binding, although it is possible that IGFBPs can also potentiate the local activity of IGFs through binding to cell-surface proteoglycans and local ECM and thus locally concentrate IGFs and increase their paracrine and/or autocrine signaling (53). Moreover, it is thought that IGFBPs have their own IGF-independent actions, either binding to cell-surface proteins that may act as IGFBP receptors or directly translocating to the nucleus and affecting gene transcription (54). The present study has shown that IGFBP2, -3, -5 and -6 levels are differentially regulated after TGF-β stimulation in the absence of RGC-32. IGFBP2 is the most abundant member of this family in the CNS, being expressed by astrocytes, neurons, and cerebral blood vessels (49). Chesik et al. have shown that reactive astrocytes from MS lesions display an increased IGFBP2 reactivity, and *in vitro* treatment of cultured astrocytes with IGFBP2 and IGF-1 enhances their proliferation, suggesting that in MS, IGFBP2 may serve to enhance reactive astrogliosis (55).

One interesting finding was that EZR was highly up-regulated in WT astrocytes, in contrast to RGC-32 KO astrocytes. EZR is a member of the ezrin–radixin–moesin (ERM) family of proteins and interacts with membrane receptors such as CD44, which was also upregulated in WT astrocytes. Interestingly, the EZR-CD44 interaction has been found to play a role in morphological changes and the motility of astrocyte processes (56). Their differential regulation in the absence of RGC-32 might explain, in part, the morphological differences that we have observed between WT and KO astrocytes both *in vitro* and during EAE.

We found that the expression of CTGF is impaired in cultured astrocytes stimulated with TGF-β and in spinal cord astrocytes from mice with EAE in the absence of RGC-32. This growth factor, also known as CCN2, belongs to the CCN family of matricellular proteins and has domain homology to the IGFBPs (57). Dysregulation of CTGF signaling has been found to play a major role in pathological processes such as fibrosis, where, for example, CTGF acts as a downstream mediator of TGF-β effects on ECM synthesis (58). Increased levels of CTGF have been detected in both astrocytes and neurons in the brains of patients with MS, Alzheimer’s disease, and amyotrophic lateral sclerosis, as well as in glioblastoma (59). A recent study by Lu and coworkers has shown that activation of cultured astrocytes by CTGF results in increased expression of inflammatory cytokines and chemokines as well as increased chemotaxis of inflammatory cells (41). The same authors found that CTGF released by activated astrocytes can act in an autocrine and paracrine manner to amplify the inflammatory response (41). Intriguingly, a study by Mendes et al. has shown that CTGF is also involved in astrocyte differentiation (40). We have previously demonstrated that the expression of ECM components is regulated by RGC-32 in cultured astrocytes (25, 60). We now show for the first time that RGC-32 acts upstream of CTGF in the TGF-β signaling pathway, and RGC-32-mediated regulation of CTGF expression is involved in ECM production.

In conclusion, the significant role played by RGC-32 in the differentiation of both Th17 cells (23) and astrocytes makes this molecule a new and reliable therapeutic target in both relapsing-remitting and secondary progressive MS.

## Data Availability Statement

The raw data supporting the conclusions of this article will be made available by the authors, without undue reservation.

## Ethics Statement

The animal study was reviewed and approved by University of Maryland School of Medicine Office of Animal Welfare Assurance.

## Author Contributions

AT, VR, and HR designed the study. AT, AB, VN, DB, AM, CC, and TB performed the experiments. AT, DFM, VR, and HR wrote the manuscript. All authors contributed to the article and approved the submitted version.

## Funding

This work was supported in part by a grant from Veterans Administration Merit Award (I01BX001458 to HR) and by an RO1 NS42011 grant (to HR).

## Conflict of Interest

The authors declare that the research was conducted in the absence of any commercial or financial relationships that could be construed as a potential conflict of interest.

## Abbreviations

BBB, blood–brain barrier; CNS, central nervous system; CTGF, connective tissue growth factor; EAE, experimental autoimmune encephalomyelitis; ECM, extracellular matrix; EZR, ezrin; FABP7, fatty acid binding protein 7; GFAP, glial fibrillary acidic protein; IGF1, insulin-like growth factor 1; IGF1R, IGF1 receptor; IGFBP, insulin-like growth factor binding protein; KO, knock-out; MMP, matrix metalloproteinase; MS, multiple sclerosis; PLAUR, plasminogen activator, urokinase receptor; OD, optical density; RGC-32, response gene to complement 32; STAT3, signal transducer and activator of transcription 3; VEGF, vascular endothelial growth factor; WT, wild type.
