# Supplementary material for: RGC-32 Regulates Generation of Reactive Astrocytes in Experimental Autoimmune Encephalomyelitis
Source: Front Immunol. 2021 Jan 25;11:608294. doi: 10.3389/fimmu.2020.608294 (PMC7868332; doi:10.3389/fimmu.2020.608294)
Supplement: Supplementary file 1 [file DataSheet_1.pdf]

Supplementary Material

1. Tables

Supplementary table | Mouse primers used for Real Time PCR

Gene symbol	Primers sequence
CTGF	For: 5'- CCTGCCCTAGCTGCCTAC -3'<br>Rev: 5'- GCACTTTTTGCCCTTCTTAA -3'
IGF1	For: 5'- GCTGCTGAAGCCATTCATTTAG -3'<br>Rev: 5'- CGTGGGAAGAGGTGAAGATAAG -3'
IGFBP2	For: 5'- GGTGCCAAACACCTCAGTCT -3'<br>Rev: 5'- GGAGATCCGCTCCAGGAC -3'
IGFBP3	For: 5'- GCAGCCTAAGCACCTACC -3'<br>Rev: 5'- GCTTAGACTGCCAGGAGAAGTTC -3'
MMP2	For: 5'- GTTCAACGGTCGGGAATACA -3'<br>Rev: 5'- GCCATACTTGCCATCCTTCT -3'
MMP9	For: 5'- CTGGAACTCACACGACATCTT -3'<br>Rev: 5'- TCCACCTTGTTCACCTCATTT -3'
18S	For: 5'- GTAACCCGTTGAACCCCATT -3'<br>Rev: 5'- CCATCCAATCGGTAGTAGCG -3'

2. Figures

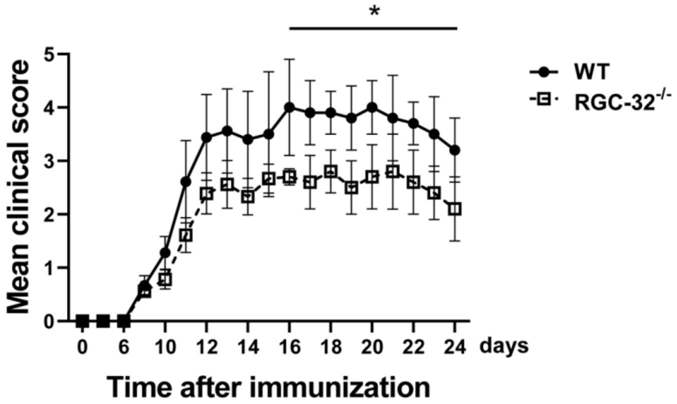

Supplementary Figure 1. Lack of RGC-32 attenuates the clinical course of EAE

WT and RGC-32 KO mice were immunized with $MOG_{35–55}$ and then clinical scores were determined during EAE as described in Materials and Methods. Data are expressed as mean ± SEM of clinical EAE scores and are representative of three independent experiments (6 mice for each group/ experiment). * = p<0.05.

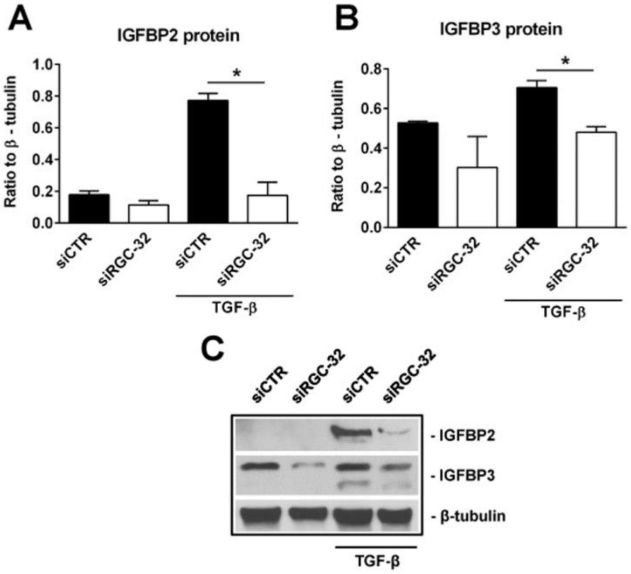

Supplementary Figure 2. RGC-32 silencing affects the expression of IGFBP2 and IGFBP3 proteins in rat astrocytes

Cultured rat astrocytes were transfected with siRGC-32 or siCTR using Lipofectamine 3000, then stimulated with TGF-β (10 ng/ml) for 24 h. RGC-32 silencing significantly reduced the protein expression of IGFBP2 (**A**) and IGFBP3 (**B**) when compared with that of siCTR. The protein expression was normalized to β-tubulin. A representative blot for each protein is shown in **C**. Results are expressed as mean ± SEM (n=3). * = p<0.05.

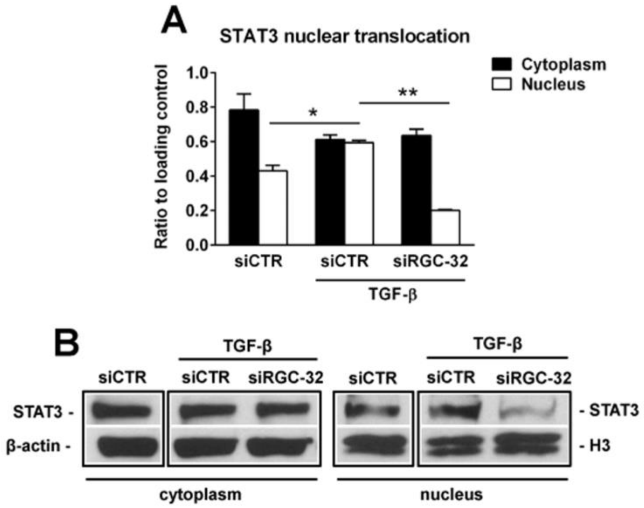

Supplementary Figure 3. STAT3 nuclear translocation depends on RGC-32

Primary rat astrocytes were transfected with siRGC-32 or siCTR using Lipofectamine 3000, then stimulated with TGF-β (10 ng/ml) for 4 h. Cell lysates were separated into cytoplasmic and nuclear fractions and then analyzed by Western blotting. TGF-β treatment resulted in a significant increase in STAT3 nuclear translocation, and this process was greatly reduced when RGC-32 was silenced. The results are expressed as ratios to β-actin for the cytoplasmic fraction and to histone H3 for the nuclear fraction. Representative blots are shown in **B**. Data in **A** are shown as mean ± SEM (n=3). * = $p<0.05$; ** = $p<0.01$.
